# Mediterranean Recluse Spider, *Loxosceles rufescens* (Araneae: Sicariidae) from Charkhab Cave, Southern Iran

**Published:** 2017-03-14

**Authors:** Saber Sadeghi, Meysam Dashan, Mohammad Javad Malek-Hosseini

**Affiliations:** Department of Biology, Faculty of Sciences, Shiraz University, Shiraz, Iran

**Keywords:** Arachnida, *Loxosceles*, Iran

## Abstract

**Background:**

The best-known dangerous spiders belong to the six genera. The genus *Loxosceles* or violin spiders are well known for their ability to cause skin necrosis or loxoscelism. All *Loxosceles* species have medical importance due to their necrotizing venom. The present article reports the occurrence of *L. rufescens* in Charkhab Cave, south of Iran (Larestan).

**Methods:**

The specimens were collected from the Charkhab Cave using handling forceps, paintbrush and aspirator and preserved in 96% ethanol.

**Results:**

*Loxosceles rufescens*, a medically important spider, is recorded from Charkhab Cave in Fars Province (southwest of Iran). Identification of *L. rufescens* was performed based on external morphology and the features of male genitalia.

**Conclusion:**

Presence of *L. rufescens* in south of Iran especially in a cave confirmed that this species is a widely distributed species in Iran. Therefore, cavers or cave visitors should be aware of this poisonous spider in caves.

## Introduction

Spiders with more than 45,000 described species in the world (Shahi et al. 2013) are among the better-studied arthropods. However, approximately 200 species from 20 genera of worldwide spiders can cause severe human injuries, with dermonecrosis, systemic toxicity, and death. Therefore, less than 0.5% of them are actually dangerous for humans (Diaz 2004). The most known dangerous spiders belong to the six genera: *Latrodectus* (black widow spiders), *Steatoda* (false widow spiders), *Loxosceles* (recluse spiders), *Atrax* (funnelweb spiders), *Phoneutria* (banana spiders), *Cheiracanthium* (foliage spiders) (Yigit et al. 2008).

The recluse spiders (Sicariidae: *Loxosceles*) or Mediterranean Fiddle-Back spiders are cosmopolitan, but most commonly in the tropics. Although previously placed in the family Loxoscelidae (Gertsch 1949, Gertsch and Ennik 1983) or Scytodidae (Gertsch 1967), Sicariid spiders are now considered a member of family Sicariidae (Plantick et al. 1991). *Loxosceles rufescens* (Dufour 1820) occurs around the entire Mediterranean region, in Asia, North Africa, South and Central America (Platnick 2013). Several species (troglophile) have been recorded from caves, houses, subterranean areas, rural and urban area (Fischer et al. 2009).

Although some unidentified species of the genus *Loxosceles* reported from Iran during 1994 to 2013 (Goodarzi 1994, Moradmand and Jäger 2011, Kashefi et al. 2013, and Mirshamsi et al. 2013), the first record of Mediterranean recluse spider, *L. rufescens*, in Iran was presented by Zamani and Rafinejad (2014) from Tehran Province. Shahi et al. 2013 also reported a Loxoscelism from Bandar Abbas in south of Iran. All *Loxosceles* species have medical importance due to their necrotizing venom, based on Swanson and Vetter study (2006) “Spiders of the genus *Loxosceles* cause necrotic dermatologic injury through a unique enzyme, sphingomyelinase D, found only in one other spider genus and several bacteria”. Tambourgi and et al. (2000) also noted, **“**Envenomation causes dermonecrosis and complement (C)-dependent intravascular hemolysis”**.** Typical symptoms start 2–6 h after the bite and the reactions to bites can be variable from mild to severe, and occasionally leads to death. “Symptoms can include fever, rash, nausea, vomiting, hemolytic anemia, bloody urine, renal failure, and shock” (Hufford 1977).

In Iran, The Araneae fauna of Iranian caves has been poorly studied. Marusik et al. (2014), published a survey of spider species from Shirabad Cave, Golestan Province, north of Iran, and the first troglobite species of Iranian spiders *Trilacuna qarzi* Malek Hosseini and Grismado, 2015, was reported by Malek Hosseini et al. (2015). Some sporadic studies such as Moradmand and Jäger (2011) and Zamani et al. (2014, 2015) have been conducted so far.

The present contribution reports the occurrence of *L. rufescens* in Charkhab Cave in south of Iran (Larestan). It may help to cover a very small part of information gap of our knowledge of the situation of this important genus in Iran and aware caverns about *L. rufescens* presence in this cave and possibly other caves.

## Materials and Methods

### Study area

**Fig. 1.**
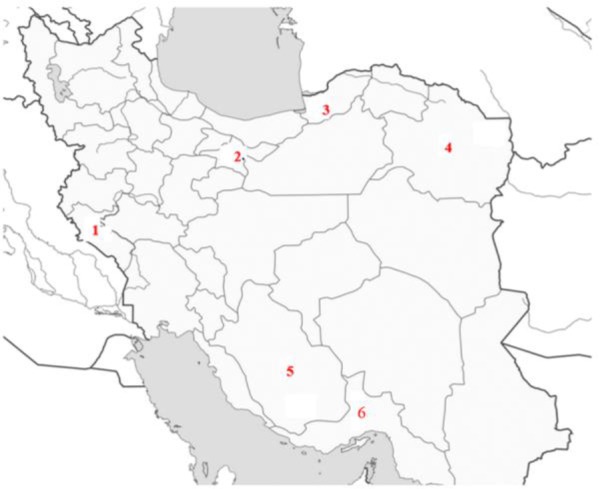
Sampling localities of genus *Loxosceles* and species *Loxosceles rufescens* in Iran reported up to 20151: Ilam Province, (*Loxosceles* sp) Moradmand and Jäger 2011.2: Tehran Province (35°43'N, 51°25′E), Zamani and Rafinajad 2014.3: Golestan Province (36°50′19″N, 54°26′05″E), Kashefi et al. 2013.4: Khorasan Province (36°16′24 95″N, 59°34′ 75″E), Mirshamsi et al. 2013.5: Fars Province, Laresthan (27° 32′ 44″N, 55° 20′ 21″E), present study.6: Hormozgan Province, Bandar Abbas (27 ° 11′ 0″N, 56°16′36.0″E), Shahi et al. 2013.

Charkhab Cave is in Larestan area, south of Fars Province, 70 km south of Larestan (Fig. 1) in Hormoud protected area, near villages of Zad Mahmood and Ahveh with warm and dry climate surrounded by mountains (27° 32′ 44″ N: 55° 20′ 21″ E), altitude: 639 m a.s.l. This cave is one of 51 explored caves in Fars with valuable habitants especially spider species. The cave was visited on Jan. 8, 2014.

### Collection and Identification

The walls and floor of the cave were precisely searched for cavernicoles. The specimens were collected by authors during a fieldtrip on January 2014 using common methods such as nets, forceps and soft paintbrush then, they preserved in 96% Ethanol and transported to our Entomology Lab. in Shiraz University. Some photos were taken by a digital camera (Canon DS126251).

The spiders were distinguished based on external morphology and features of the male genitalia using available keys. Finally, our description was confirmed by Dr YM Marusik and Dr RS Vetter, two world specialists of the spiders. The specimens were deposited in the Zoological Museum, Collection of Biology department of Shiraz University (ZMCBSU).

## Results

Laboratory works and separating specimens from samples clarified that 5 adult males and 3 females were collected.

**Fig. 2.**
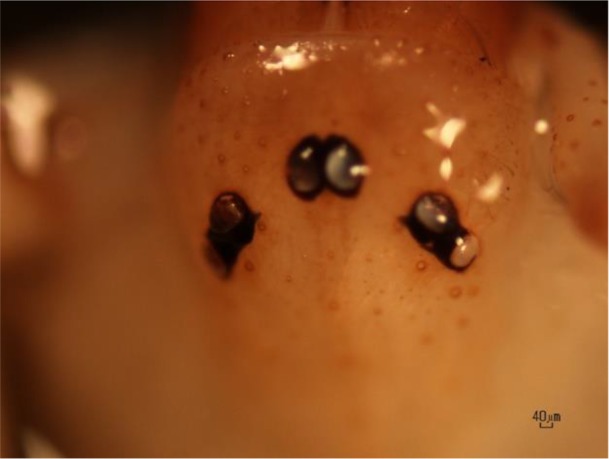
*Loxosceles rufescens*, arrangement of eyes in three pairs

*Loxosceles rufescens* or fiddle back spider unlike most other spiders that have eight eyes, has six eyes as three pairs in a nearly triangular arrangement (Fig. 2). A distinctive “violin” shaped dark pattern is present on the cephalothorax of this species. However, using this overly simplified diagnostic identifier will lead to mistakes because some *Loxosceles* species have almost no pigmentation in the violin area and other species have dark maculae on the dorsal body surfaces. A particular diagnostic character of the genus (*Loxosceles*) is existence of six eyes, which arranged in nontouching pairs in a U-shaped pattern. Most spiders have eight eyes, as a hallmark for this medically important group.

**Fig. 3.**
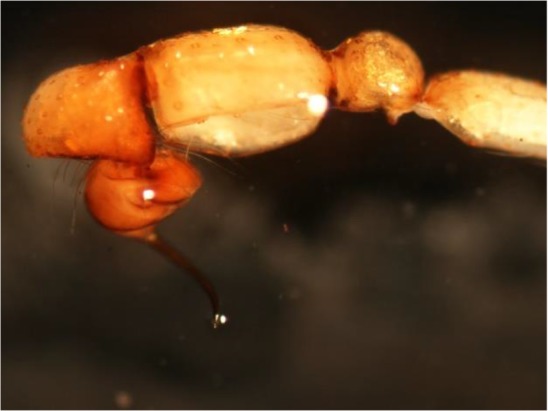
*Loxosceles rufescens,* male palpus, lateral view

The tibia of males’ palpus is short, thick, and narrow at base and not very prolonged (Fig. 3). The embolus length is about as long as the width of the globular bulb.

## Discussion

**Fig. 4.**
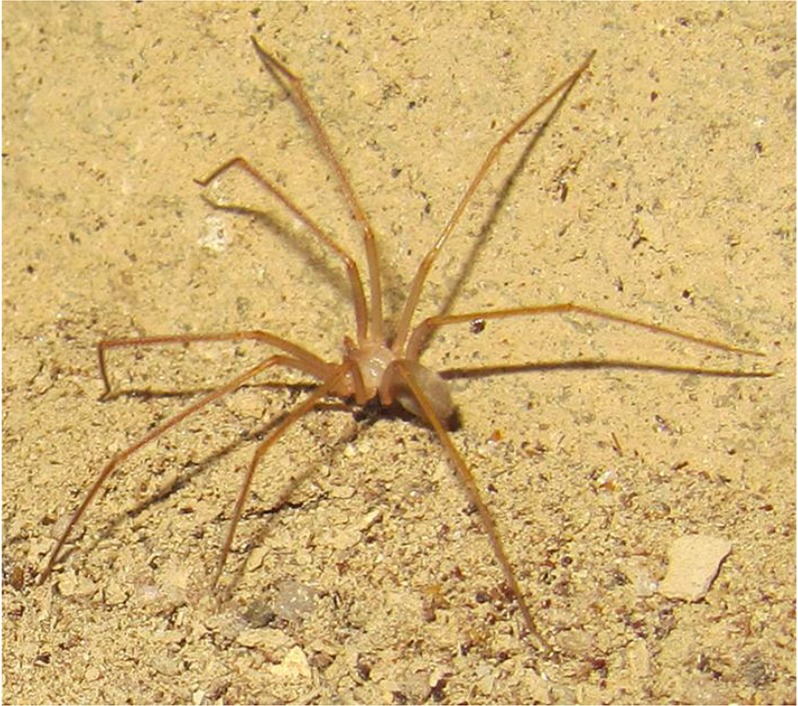
*Loxosceles rufescens*, male, Charkhab Cave

The Loxoscelinae is a small subfamily with about 120 species in a single genus. *Loxosceles rufescens* (Fig. 4) is native to the Europe and North Africa and then spread to other regions by human activity. It occurs in Mediterranean area and Middle East to western Russia, besides has been introduced to Madagascar, southern Asia, Australia, Atlantic and Pacific Islands. In north and south of America, they have also been reported from many states (Green et al. 2009).

Some species have also been introduced into human habitations, where they have been able to establish permanent populations. Although, this species have been reported from some parts of Iran (Mirshamsi et al. 2013, Zamani and Rafinejad 2014) but the present report is the first record of the species from an Iranian cave. This cave has recently been discovered and is habitat of many bats, a kind of snake (not captured) and a variety of arthropods. *Loxosceles rufescens* was observed throughout the cave especially at the hypogean part. This species have only been collected from Charkhab Cave up to now, it probably is partly due to high temperature and dry weather of the area. However, it does not mean that this species cannot spread to other caves. Therefore, cavers or cave visitors should be aware of potential dangers of the poisonous spiders in caves because there is no evidence-based effective therapy for loxoscelism at present time.

## Conclusion

The presence of *L. rufescens* in a cave in south of Iran confirms that this species is also a cave adapted species in Iran, but not distributed widely because we found it only in one of more than seventeen investigated caves by authors and their collaborators. However, cavers and other visitors of caves have to take care because of some dangerous spiders with poisonous venom. Our collected specimens were morphologically a little different from other areas of the world thus more studies are needed to reveal their taxonomic and toxicological differences.
